# Effect of Preoperative Immunonutrition on Postoperative Major Morbidity after Cytoreductive Surgery and HIPEC in Patients with Peritoneal Metastasis

**DOI:** 10.3390/nu13072147

**Published:** 2021-06-23

**Authors:** Alba Fernández-Candela, Alicia Calero, Luís Sánchez-Guillén, Javier Escrig-Sos, José A. Barreras, Francisco López-Rodríguez-Arias, Laura Armañanzas, Ana Murcia, Antonio Arroyo, Francisco Javier Lacueva

**Affiliations:** 1 Peritoneal Carcinomatosis Unit, General Surgery Department, Elche University General Hospital, 03202 Elche, Spain; albafmed@gmail.com (A.F.-C.); drsanchezguillen@gmail.com (L.S.-G.); jabmelx@gmail.com (J.A.B.); franloarias@hotmail.com (F.L.-R.-A.); laura.armananzas@gmail.com (L.A.); arroyocir@yahoo.es (A.A.); fj.lacueva@gmail.com (F.J.L.); 2 Department of Medicine, University Jaume I (UJI), 12004 Valencia, Spain; fj.lacueva@umh.com; 3 Pathology and Surgery Department, Universidad Miguel Hernandez, 03202 Elche, Spain; 4 Pharmacy Department, Elche University General Hospital, 03202 Elche, Spain; murcia_ana@gva.es

**Keywords:** peritoneal metastasis, peritoneal carcinomatosis, cytoreductive surgery with hyperthermic intraperitoneal chemotherapy (CRS-HIPEC), immunonutrition, postoperative complications, C-reactive protein

## Abstract

The effect of preoperative immunonutrition intake on postoperative major complications in patients following cytoreductive surgery (CRS) with or without hyperthermic intraperitoneal chemotherapy (HIPEC) was assessed. The accuracy of C-Reactive Protein (CRP) for detecting postoperative complications was also analyzed. Patients treated within a peritoneal carcinomatosis program in which a complete or optimal cytoreduction was achieved were retrospectively analyzed. They were divided into two groups based on whether preoperative immunonutrition (IMN) or not (non-IMN) were administered. Clinical and surgical variables and postoperative complications were gathered. Predictive values of major morbidity of CRP during the first 3 postoperative days (POD) were also evaluated. A total of 107 patients were included, 48 belonging to the IMN group and 59 to the non-IMN group. In multivariate analysis immunonutrition (OR 0.247; 95%CI 0.071–0.859; *p* = 0.028), and the number of visceral resections (OR 1.947; 95%CI 1.086–3.488; *p* = 0.025) emerged as independent factors associated with postoperative major morbidity. CRP values above 103 mg/L yielded a negative predictive value of 84%. Preoperative intake of immunonutrition was associated with a decrease of postoperative major morbidity and might be recommended to patients with peritoneal carcinomatosis following CRS. Measuring CRP levels during the 3 first postoperative days is useful to rule out major morbidity.

## 1. Introduction

Until 20 years ago, peritoneal carcinomatosis from gastrointestinal cancers was considered a terminal stage and these patients received only palliative treatment. However, the development of cytoreductive surgery (CRS) alone or in combination with hyperthermic intraperitoneal chemotherapy (HIPEC) has been increasingly introduced to treat peritoneal metastasis of some neoplasms with curative intent [1,2]. Nowadays, it is considered the standard of care to treat peritoneal pseudomyxoma and mesothelioma while CRS alone or in combination with HIPEC has also been shown to improve overall and disease-free survival in peritoneal carcinomatosis of ovarian and colonic origin [3,4,5]. Nevertheless, the effectivity of HIPEC on these neoplasms is still a matter of debate [6,7].

Major morbidity is a critical issue in the CRS and HIPEC procedure, reported to be around 30% [8]. Even so, these figures are consistent with those reported with other major surgical procedures [9,10] and they are mainly associated with the extent of the radical surgery performed [8]. In particular, peritoneal carcinomatosis index (PCI), number of visceral resections and digestive anastomoses and the extension of peritonectomies stand out as independent predictors of postoperative complications [8,11,12]. Postoperative major morbidity constitutes not only a life-threatening side effect but may also jeopardize long-term survival and quality of life [13,14].

Therefore, there is a growing interest in developing preoperative strategies in the setting of pre-habilitation protocols to diminish postoperative complications. Surgical stress is known to produce an acute depletion in arginine levels that impair T lymphocyte function and nitric oxide synthesis, increasing the risk of infection as well as impairing wound healing [15]. Perioperative immunonutrition including arginine, glutamine, omega 3 and nucleotides among other nutrients provides a postoperative reduction of inflammation markers such as C-reactive protein (CRP), TNFa and endotoxin [16]. Moreover, these immuno-nutrients may enhance protein synthesis after surgery reducing septic and other postoperative complications [17]. Some studies have shown that the intake of immunonutrition may decrease overall postoperative infectious and non-infectious complications in patients undergoing major oncological surgery [18,19]. However, this benefit has been questioned in some other studies [20,21], and has not been studied so far in patients with peritoneal carcinomatosis following CRS procedures.

Early detection and treatment of postoperative complications is also an important issue to decrease mortality rates in these patients and some inflammatory markers have been assessed for this purpose. Among them, CRP serum levels monitored at the early postoperative period seem more accurate than other markers such as procalcitonin and leukocyte count to detect septic complications at the postoperative period [22,23,24].

The aim of our study was to assess the effect of preoperative immunonutrition on postoperative major complications in patients with peritoneal carcinomatosis following CRS procedures with or without HIPEC. The usefulness of CRP levels as a tool to detect postoperative major complications was also analyzed.

## 2. Materials and Methods

### 2.1. Study Design and Patient Selection

All patients treated for peritoneal carcinomatosis within a peritoneal carcinomatosis program at the Elche University General Hospital from November 2014 to November 2020 were initially considered. Finally, only those patients in whom a complete or optimal cytoreduction was achieved after CRS with or without HIPEC were included in the study. Written informed consent was obtained from all patients before surgery.

A retrospective cohort study was carried out from a prospective database of these patients. Two groups based on whether they had received immunonutrition supplements (IMN) at the preoperative period or not (non-IMN) were compared. This study was approved by the Ethics Committee of our institution (PI 21/2018).

### 2.2. Preoperative and Surgical Management

Nutritional assessment was performed 3 to 4 weeks before surgery. A varied diet rich in protein was recommended to all patients according to their preference in food intake. Immunonutrition supplements were administered twice daily to the IMN group with Atempero^®^ 200 mL every 12 h during 7 days prior to surgery. Atempero^®^ is a hyper-proteic diet including immuno-nutrients such as L-arginine, omega-3 fatty acids and nucleotides. Immunonutrition supplements were not prescribed before October 2018 but they were administered thereafter. All patients were admitted to the hospital the day before surgery and antibiotic and antithrombotic prophylaxis was administered according to protocol.

The peritoneal tumor burden was quantified according to the PCI and the degree of cytoreduction was assessed at the end of the surgical procedure according to the Cytoreductive Completeness Score (CC) [25]. Drugs used during the HIPEC procedure were Mitomycin-C and Oxaliplatin in patients with peritoneal metastasis from colorectal origin or peritoneal pseudomyxoma. Oxaliplatin was only administered to 8 patients and since 2016 has not been used anymore. Paclitaxel was administered in women with ovarian peritoneal metastasis. HIPEC was initially delivered with an open-coliseum technique but since January 2018 it has been delivered with a closed technique (PRS Combat, Galmaz Biotech, Madrid, Spain).

### 2.3. Outcome Measures

Data including demographic characteristics such as age and sex, as well as American Society of Anesthesiologists (ASA) classification, carcinomatosis origin, neoadjuvant chemotherapy and immunonutrition intake, were gathered. Adherence to treatment with immunonutrition supplements was verified by asking each patient the day before surgery. Surgical variables such as the number of visceral resections, PCI and the administration of HIPEC were also recorded. The PCI was categorized as low (1–5), medium (6–15) and high (>15). The 30-day and in hospital postoperative complications were registered according to the Clavien-Dindo classification [26], and subsequently graded as none, minor (I-II) and major (III-V). In addition, the occurrence of digestive leak and the need for reoperation were also recorded. In-hospital length of stay was also included.

Complete blood count and blood chemistry including albuminemia and CRP levels were obtained no longer than 3 weeks prior to surgery. Subsequently, a complete blood count and CRP levels were obtained during the first 3 postoperative days (POD).

### 2.4. Statistical Analysis

Continuous variables were described with the median and the interquartile range. Categorical variables were described with frequencies and percentages. The Mann-Whitney test was used for the comparison of medians, and the Chi-square test for the comparison of proportions. Control of potential confounders regarding the risk of developing major complications was performed using a Binary Logistic Regression (IBM SPSS Statistics for Windows, version 25.0. IBM Corp.: Armonk, NY, USA). The statistical significance was set at *p* < 0.05.

To assess the evolution of CRP values in patients with and without postoperative major morbidity, a linear general model (repeated measures ANOVA) was used. A non-parametric receiver operating characteristic (ROC) curve was performed to describe sensitivity, specificity, positive predictive value and negative predictive value of CRP as a predictor of major morbidity and to determine the most discriminating cut-off value.

## 3. Results

In total, 107 patients with peritoneal metastasis that underwent a CRS with or without HIPEC were included in our analysis. Of these, 48 received immunonutrition supplements preoperatively and 59 did not. Baseline features are shown in Table 1. There were no differences between the two groups in the distribution of age, sex and ASA score. IMN patients received neoadjuvant therapy more frequently (*p* = 0.047). Ovarian and colorectal carcinomas were the most frequent origin but, while prevalence of ovarian and colorectal carcinomatosis was similar in the non-IMN group (40.7% and 37.3%), ovarian carcinomatosis was more prevalent (60.4%) in the IMN group.

### 3.1. Surgery

Surgical data are shown in Table 1. PCI showed a trend to be higher in the IMN group as compared with the non-IMN group attending to median (10 vs. 8) and categories (*p* = 0.077), although statistical significance was not reached.

More visceral resections were performed in the IMN group in comparison with the non-IMN patients (*p* = 0.002). Thus, while none or only one visceral resection was performed in 64% of the non-IMN patients, 73% of the patients belonging to the IMN group underwent two or more visceral resections. 

On the contrary, HIPEC was less frequently administered to IMN patients (68.8% vs. 89.8%; *p* = 0.006). Finally, a complete cytoreduction score (CC0) was achieved in 88% of the patients in both groups.

### 3.2. Immunonutrition and Postoperative Complications

Overall, 28 (26.2%) patients presented major morbidity (Clavien III-V). Of these, only 11 (39.3%) were infectious complications. Postoperative major complications occurred more frequently in the non-IMN group patients compared to the IMN group (30.5% and 20.8%, respectively) although this difference did not reach statistical significance. Digestive leak occurred more frequently in the IMN group (14.6% vs. 8.5%) but the reoperation rate was similar in both groups (8.3% vs. 10.2%). Thirty-day or in-hospital postoperative mortality (Clavien V) was 1.9%. The two postoperative deaths occurred in the non-IMN group due to an oxaliplatin adverse event and to a respiratory failure after a reoperation. Postoperative complications are summarized in Table 2.

With regard to postoperative major complications, median PCI (*p* = 0.012) and number of visceral resections (*p* = 0.006) were related to the onset of major morbidity (Table 3). Immunonutrition, age, ASA classification, neoadjuvant treatment, preoperative CRP, PCI, number of visceral resections, and HIPEC administration were subsequently included in the multivariate analysis, and only two of these arose as independent predictive factors (Table 4). Thus, immunonutrition emerged as an independent protective factor (OR 0.247; 95%CI 0.071–0.859; *p* = 0.028) while the number of visceral resections arose as an independent risk factor (OR 1.947; 95%CI 1.086–3.488; *p* = 0.025) against developing postoperative major morbidity. The posterior analysis of the two statistically significant variables showed a power of 97% for immunonutrition and 88% for number of visceral resections.

### 3.3. CRP Values

Preoperative CRP serum levels were available in 95 (88.8%) patients. CRP values below 10 mg/L were found in 82.1% of the patients while only 9.5% of the patients had values above 20 mg/L. All the patients with low PCI score showed CRP values lower than 10 mg/L.

**Figure 1:**
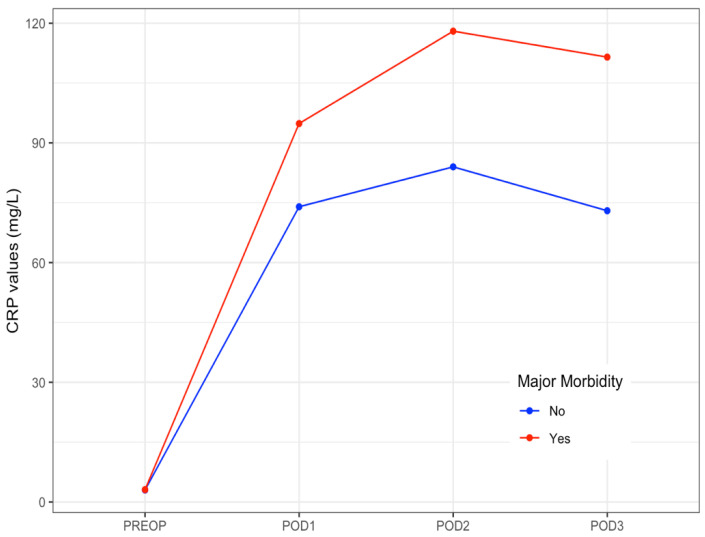
Daily median levels of CRP in patients with and without postoperative major morbidity. Evolution at the early postoperative period. CRP: C-Reactive Protein. PREOP: Preoperative. POD: postoperative day.

**Figure 2:**
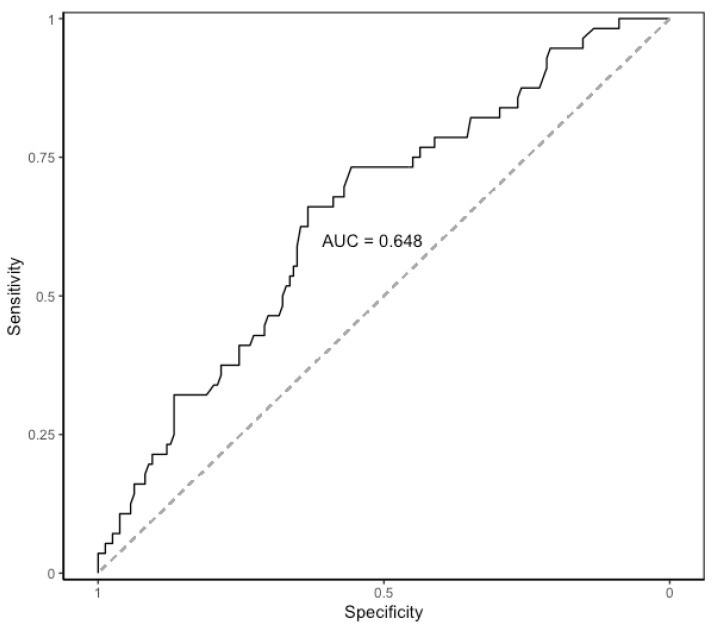
Area under the ROC curve of CRP on postoperative days 2 and 3. AUC: area under the ROC curve. ROC: receiver operating characteristic. CRP: C-Reactive Protein.

CRP levels raised on POD 1, peaked on POD 2, and then slightly decreased on POD 3. The median values of CRP on POD 1 to POD3 are shown in Table 5. The evolution of median levels of CRP belonging to the patients exhibiting postoperative major complications was different to those not doing so (*p* = 0.007) (Figure 1). The area under the ROC curve obtained on POD 2 and POD 3 was 0.65 (95%CI 0.58–0.71). The optimal cut-off CRP value was 103 mg/L, yielding a sensitivity of 66%, a specificity of 63%, a positive predictive value of 39%, and a negative predictive value of 84% (Figure 2).

## 4. Discussion

To our knowledge, this is the first study to evaluate the effect of preoperative immunonutrition intake on postoperative complications in patients with peritoneal carcinomatosis. Our analysis showed that the preoperative intake of immunonutrition was an independent protective factor against severe morbidity, after taking into consideration that IMN patients exhibited a higher PCI and underwent more visceral resections compared to non-IMN patients. This fact is relevant because PCI and the number of visceral resections were significantly associated with the occurrence of major complications at the early postoperative period. The correlation of PCI and the need for more extensive surgical procedures carrying higher morbidity have already been previously reported [8,12,27].

Neoadjuvant chemotherapy was administered more frequently to the IMN patients while HIPEC was administered more frequently in the non-IMN group. None were associated with major morbidity, although one patient of the non-IMN group died due to oxaliplatin related toxicity administered at the HIPEC procedure. Major morbidity related to oxaliplatin, especially regarding postoperative hemorrhage, has been shown in the PRODIGE trial [7]. Nevertheless, overall neither HIPEC nor neoadjuvant chemotherapy significantly increases major morbidity over CRS alone [8].

Patients included in our study did not follow a specific pre-habilitation program that might confound with the effect of immunonutrition alone on postoperative complications. In this sense, the combination of enhanced recovery after surgery (ERAS) with perioperative immunonutrition supplements has been shown to reduce postoperative complications after colorectal surgery [28]. On the other hand, we assessed the protective effect of immunonutrition against major morbidity according to the Clavien-Dindo classification, hence infectious and non-infectious severe complications were included. Some recent studies assessed the effect of preoperative or perioperative immunonutrition on postoperative complications, showing conflicting results. Thus, while a reduction in infectious and non-infectious complications was found in two systematic reviews and meta-analysis [18,19], this protective effect was not found in a large national cohort study [21]. It should be emphasized that these studies included patients with different gastrointestinal malignancies following surgical procedures of variable extent. Nowadays, the intake of enteral immunonutrition in the perioperative setting of patients undergoing radical surgery for digestive cancers is advocated in some countries, but there is no consensus with regard to the recommendations among different medical societies [29]. Extensive CRS is usually performed in patients with peritoneal malignancies entailing a remarkable inflammatory response and loss of proteins, and the effect of immunonutrition could hypothetically be especially beneficial in these patients. Thus, the inclusion of immuno-modulatory supplements into pre-habilitation programs for patients with peritoneal malignancies undergoing CRS and HIPEC has been recently advocated [30].

In all patients, CRP levels peaked on POD 2, and then slightly decreased on POD 3 as has been previously reported [22,31], but median levels of CRP belonging to the patients with postoperative major complications were significantly higher. We found also that the cut-off CRP value yielded a good negative predictive value in ruling out major morbidity but the sensitivity and specificity were poor. The interest in the high negative predictive value of CRP obtained at the early postoperative period has already been described in patients following colorectal surgery, which has prompted proposed CRP as a useful marker to rule out anastomotic leak or infectious complications [23,24]. Moreover, CRP values on POD 4 seem to provide the best accuracy for predicting infectious complications [22,31], but unfortunately CRP values at POD 4 were missing in one-third of our patients. Recently, a study carried out on patients with peritoneal carcinomatosis following CRS and HIPEC analyzed the CRP values at the early postoperative period reporting a cut-off CRP value higher than that found in our series [32]. In this report, however, complications were classified according to the SAE grading system and the proportion of infectious complications was higher compared to that observed in our study.

The main limitation of our analysis lies in the comparison of two retrospective cohorts over two consecutive periods of time. Although a learning curve bias might thus be argued, CRS was performed by the same the senior surgeons following the same protocols in both periods, and the complete CRS rate achieved and the reoperation rate were similar in IMN and non-IMN patients. Only the technique for delivering HIPEC has changed since 2018 from the open-coliseum technique to a closed technique, but the HIPEC procedure in itself was not associated with different major morbidity rates.

In conclusion, our study suggests that preoperative administration of immunonutrition supplements is a protective factor against postoperative major morbidity in patients with peritoneal carcinomatosis following CRS with or without HIPEC. Therefore, it might be eventually recommended in this setting, although this result should be confirmed in a prospective randomized trial. Measuring of CRP levels during the early postoperative period may be useful in ruling out major morbidity in these patients.

## Figures and Tables

**Table 1:** Baseline and surgical characteristics.

	Overall	No Immunonutrition	Immunonutrition	*p*
	*n* = 107	*n* = 59	*n* = 48	
Age	60 (54–68)	60 (52–68)	61 (55–67)	0.329
Sex				0.26
Men	28 (26%)	18 (31%)	10 (21%)	
Women	79 (74%)	41 (69%)	38 (79%)	
ASA score				0.579
I	10 (9.3%)	7 (11.9%)	3 (6.3%)	
II	63 (58.9%)	33 (55.9%)	30 (62.5%)	
III	34 (31.8%)	19 (32.2%)	15 (31.3%)	
Neoadjuvant therapy				0.047
No	51 (48.1%)	33 (56.9%)	18 (37.5%)	
Yes	55 (51.9%)	25 (43.1%)	30 (62.5%)	
Carcinomatosis				0.037
Ovarian	53 (49.5%)	24 (40.7%)	29 (60.4%)	
Colorectal	32 (29.9%)	22 (37.3%)	10 (20.8%)	
Pseudomyxoma	13 (12.1%)	9 (15.3%)	4 (8.3%)	
Gastric	2 (1.9%)	2 (3.4%)	0	
Endometrial	2 (1.9%)	2 (3.4%)	0	
Mesothelioma	1 (0.9%)	0	1(2.1%)	
Primary	1 (0.9%)	0	1 (2.1%)	
Others	3 (2.8%)	0	3 (6.3%)	
PCI median	8 (6–15)	8 (5–13)	10 (6–15)	0.157
PCI				0.077
1–5	24 (22.4%)	18 (30.5%)	6 (12.5%)	
6–15	63 (58.9%)	32 (54.2%)	31 (64.6%)	
>15	20 (18.7%)	9 (15.3%)	11 (22.9%)	
Visceral resections (median)	2 (1–2)	1 (0–2)	2 (1–3)	0.015
Number visceral resections				0.002
0	20 (18.7%)	15 (25.4%)	5 (10.4%)	
1	31 (29%)	23 (39%)	8 (16.7%)	
2	34 (31.8%)	12 (20.3%)	22 (45.8%)	
3 or more	22 (20.6%)	9 (15.3%)	13 (27.1%)	
Cytoreduction				0.92
Complete (CC0)	94 (87.9%)	52 (88.1%)	42 (87.5%)	
Optimal (CC1)	13 (12.1%)	7 (11.9%)	6 (12.5%)	
HIPEC				0.006
No	21 (20%)	6 (10.2%)	15 (31.3%)	
Yes	86 (80%)	53 (89.8%)	33 (68.8%)	

Data are presented as median (interquartile range 25–75) for continuous measures; number (%) for categorical measures. ASA: American Society of Anesthesiologists; PCI: Peritoneal cancer index; HIPEC: hyperthermic intraperitoneal chemotherapy.

**Table 2:** Postoperative outcomes.

	Overall	No Immunonutrition	Immunonutrition	*p*
	*n* = 107	*n* = 59	*n* = 48	
Clavien-Dindo classification				0.514
Grade 0–1	38 (35.5%)	22 (37.3%)	16 (33.3%)	
Grade II	41 (38.3%)	19 (32.2%)	22 (45.8%)	
Grade IIIa	12 (11.2%)	9 (15.3%)	4 (8.3%)	
Grade IIIb	7 (6.5%)	2 (3.4%)	4 (8.3%)	
Grade IVa	6 (5.6%)	5 (8.5%)	2 (4.2%)	
Grade V	2 (1.9%)	2 (3.4%)	0	
Clavien-Dindo categories				0.311
No morbidity	23 (21.5%)	14 (23.7%)	9 (18.8%)	
Minor morbidity	56 (52.3%)	27 (45.8%)	29 (60.4%)	
Major morbidity	28 (26.2%)	18 (30.5%)	10 (20.8%)	
Digestive leak				0.319
No	95 (88.8%)	54 (91.5%)	41 (85.4%)	
Yes	12 (11.2%)	5 (8.5%)	7 (14.6%)	
Colorectal		1 (1.7%)	5 (10.7%)	
Small bowel		2 (3.4%)	1 (2.1%)	
Gastric		1 (1.7%)		
Biliary		1 (1.7%)		
Pancreatic			1 (2.1%)	
Reoperation				0.746
No	97 (90.7%)	53 (89.8%)	44 (91.7%)	
Yes	10 (9.3%)	6 (10.2%)	4 (8.3%)	
Digestive leak		3 (5.1%)	3 (6.3%)	
Hemoperitoneum		1 (1.7%)	1 (2.1%)	
Evisceration		2 (3.4%)		
In-hospital stay	14 (10–19)	15 (10–19)	14 (10–20)	0.65

Data are presented as number (%) for categorical measures, and median (interquartile range 25–75) for continuous measures.

**Table 3:** Postoperative major complications.

	No Major Morbidity	Major Morbidity	*p*
	*n* = 79	*n* = 28	
Age	59 (53–66)	65 (59–72)	0.119
Sex			0.736
Men	20 (25.3%)	8 (28.6%)	
Women	59 (74.7%)	20 (71.4%)	
ASA score			0.223
I	9 (11.4%)	1 (3.6%)	
II	48 (60.8%)	15 (53.6%)	
III	22 (27.8%)	12 (42.9%)	
Neoadjuvant chemotherapy			0.816
No	37 (47.4%)	14 (50%)	
Yes	41 (52.6%)	14 (50%)	
Immunonutrition			0.181
No	41 (51.9%)	18 (64.3%)	
Yes	38 (48.1%)	10 (35.7%)	
Carcinomatosis			0.487
Ovarian	43 (54.4%)	10 (35.7%)	
Colorectal	22 (27.8%)	10 (35.7%)	
Pseudomyxoma	9 (11.4%)	4 (14.3%)	
Gastric	1 (1.3%)	1 (3.6)	
Endometrial	1 (1.3%)	1 (3.6%)	
Mesothelioma	0	1(3.6%)	
Primary	1 (1.3%)	0	
Others	2 (2.5%)	1 (3.6%)	
PCI median	8 (5–13)	11 (7–18)	0.012
PCI			0.083
1–5	20 (25.3%)	4 (14.3%)	
6–15	48 (60.8%)	15 (53.6%)	
>15	11 (13.9%)	9 (32.1%)	
Visceral resections (median)	1 (1–2)	2 (1–3)	0.007
Number visceral resections			0.006
0	18 (22.8%)	2 (7.1%)	
1	24 (30.4%)	7 (25%)	
2	27 (34.2%)	7 (25%)	
3 or more	10 (12.7%)	12 (42.9%)	
HIPEC			0.165
No	13 (16.5%)	8 (28.6%)	
Yes	66 (83.5%)	20 (71.4%)	
Preoperative CRP	3 (1–7)	3 (2–9)	0.071

Data are presented as median (interquartile range 25–75) for continuous measures, *n* (%) for categorical measures. ASA: American Society of Anesthesiologists; PCI: Peritoneal cancer index; HIPEC: hyperthermic intraperitoneal chemotherapy; CRP: C-Reactive Protein.

**Table 4:** Postoperative major complications. Binary Logistic Regression.

	OR (CI 95%)	*p*-Value
Age	1.016 (0.958–1.078)	0.597
ASA	2.192 (0.649–7.395)	0.206
Neoadjuvant chemotherapy	0.802 (0.256–2.513)	0.705
Immunonutrition	0.247 (0.071–0.859)	0.028
PCI	1.007 (0.904–1.122)	0.897
Visceral resections	1.947 (1.086–3.488)	0.025
HIPEC	1.044 (0.250–4.356)	0.953
Preoperative CRP	1.025 (0.996–1.056)	0.095

OR: Odds Ratio, CI: Confidence interval. ASA: American Society of Anesthesiologists classification. PCI: peritoneal carcinomatosis index. HIPEC: hyperthermic intraperitoneal chemotherapy. CRP: C-Reactive Protein.

**Table 5:** CPR levels at the preoperative and early postoperative period.

Day	Patients	Major Morbidity	
	Overall *n* = 107	No *n* = 79	Yes *n* = 28
Preoperative *	3 (2–7)	3 (1–7)	3 (2–9)
POD 1	79 (58–101)	74 (57–95)	95 (77–121)
POD 2	89 (63–147)	84 (59–127)	118 (92–182)
POD 3	88 (48–141)	73 (43–140)	112 (61–168)

CRP: C-Reactive Protein. POD: postoperative day. * CRP levels at the preoperative time were available in 95 patients. Values: median (interquartile range 25–75).

## Data Availability

The data presented in this study are available on request from the corresponding author. The data are not publicly available due to privacy restrictions.
